# The Roles of RNA Modifications in Regulating Chloroplast Performance and Photosynthesis Efficiency

**DOI:** 10.3390/ijms252211912

**Published:** 2024-11-06

**Authors:** Małgorzata Adamiec, Robert Luciński

**Affiliations:** Department of Plant Physiology, Institute of Experimental Biology, Faculty of Biology, Adam Mickiewicz University, ul. Uniwersytetu Poznańskiego 6, 61-614 Poznań, Poland; robert.lucinski@amu.edu.pl

**Keywords:** RNA modifications, chloroplast, photosynthesis

## Abstract

The regulation of gene expression is crucial for maintaining cellular activities and responding to environmental stimuli. RNA molecules are central to this regulatory network, influencing transcription, post-transcriptional processing, and translation. Recent advancements have expanded our understanding of RNA modifications beyond the nucleus, highlighting their impact on chloroplast function and photosynthesis efficiency. Chloroplasts, essential for photosynthesis, rely on precise genetic regulation to adapt to environmental changes. RNA modifications, such as methylation and pseudouridylation, are critical in regulating chloroplast RNA stability, processing, and translation. This review summarizes current knowledge of how RNA modifications affect chloroplast function and photosynthesis. It discusses the roles of specific RNA modifications occurring in chloroplast RNA, including N6-methyladenosine (m^6^A), 5-methylcytosine (m^5^C), and pseudouridylation, as well as the enzymes which are known to be involved in these processes. This review also explores extrachloroplastic RNA modifications that influence chloroplast function, emphasizing the importance of m^6^A and m^5^C modifications and their associated enzymes.

## 1. Introduction

The regulation of gene expression is a vital aspect of biology, as it is essential for maintaining an intricate balance between cellular activities and responses to environmental stimuli. This complex process is crucial for the proper functioning of organisms and the survival of their cells. Central to this regulatory network is a diverse group of RNA molecules that orchestrate the precise control of gene expression at multiple levels. Over the past decades, extensive research has revealed the complex roles of RNA in modulating transcription [1], post-transcriptional processing [2], and translation [3], thereby influencing the ultimate phenotype of an organism [4]. Understanding the interplay between RNA molecules and regulatory proteins is paramount in unraveling the molecular mechanisms underlying the regulation of gene expression. Advancements in high-throughput sequencing technologies, transcriptomics, and computational biology have provided unprecedented insight into the dynamic landscape of RNA-mediated gene regulation [5]. RNA modification, a dynamic process that occurs predominantly within the nucleus of plant cells, intricately influences gene expression by altering the chemical structure and functionality of RNA molecules. Initially investigated primarily in the context of nuclear RNA processing, recent advancements have broadened our understanding of the impact of RNA modification beyond the nucleus, extending its regulatory reach into organelles, such as the chloroplast, which is crucial for photosynthetic metabolism [6]. Despite the relatively compact nature of chloroplast genomes, they encode many essential components, including those critical for photosynthesis. The precise regulation of gene expression within chloroplasts is vital for maintaining optimal photosynthetic efficiency and adapting to environmental fluctuations. Emerging evidence suggests that RNA modifications exert a significant influence on proper chloroplast functioning by regulating not only the expression of nuclear genes related to chloroplast protein but also chloroplast gene expression itself, and by fine-tuning transcript abundance and functionality to meet the dynamic demands of chloroplasts [6].

This review aims to summarize current knowledge of the complex ways in which RNA modification influences chloroplast function and photosynthesis efficiency, ultimately enhancing our broader understanding of plant biology and its potential applications in agriculture and biotechnology.

## 2. Modifications of Chloroplast RNA

The modification of chloroplast RNA is a critical yet poorly understood aspect of plant molecular biology. Chloroplasts, the sites of photosynthesis, rely on complex interplays of genetic regulation to maintain their function and adapt to environmental stresses. RNA modifications may play an important role in the regulation of the stability, processing, and translation of chloroplast-encoded RNAs. Among the known modifications, RNA methylation is one of the most studied, and N6-methyladenosine (m^6^A) and 5-methylcytosine (m^5^C) modifications have been identified in chloroplast RNAs [7]. Pseudouridylation is another critical modification observed in chloroplasts. Pseudouridylation is the conversion of uridine into pseudouridine, which plays a role in the processing and translation of chloroplast rRNA, impacting most aspects of chloroplast development [8,9,10]. While a few proteins involved in these modifications have been discovered, the exact mechanisms and functional effects of many chloroplast RNA modifications are still poorly understood.

### 2.1. Chloroplast RNA Methylation

Chloroplasts play a pivotal role in acclimation processes. These factors dynamically respond to environmental cues and contribute significantly to the maintenance of functional photosynthesis. Moreover, the chloroplast has emerged as a central hub for orchestrating these responses, independent of certain regulatory factors. RNA methylation to form N 6-methyladenosine (m^6^A) is one of the most ubiquitous and extensively studied post-transcriptional modifications occurring in RNA. These modifications are dynamically installed and removed by a set of enzymes known as ‘writers’ and ‘erasers’, respectively, and recognized by ‘reader’ proteins, collectively constituting the RNA modification machinery. In chloroplasts, over 98% of transcripts undergo chemical modification to form m^6^A, which is a significantly higher proportion than that in the nuclear transcriptome (73%). Moreover, chloroplast transcripts exhibit a higher density of m^6^A sites, with approximately 4.6 to 5.8 sites per transcript, contrasting with cytoplasmic transcripts, which typically contain only 1.4 to 2.0 sites per transcript [11]. In addition, the distribution of m^6^A modifications in chloroplast transcripts differs from that observed in nuclear-derived mRNAs. Although nuclear mRNAs typically exhibit m^6^A enrichment within the 3′ UTR and in the proximity of stop codons, such enrichment is not observed in chloroplast transcripts. Instead, m^6^A peaks are evenly distributed across chloroplast transcripts, with higher methylation occurring in exons than in introns, indicating distinct regulatory mechanisms between the nucleus/cytoplasm and chloroplast systems. The consensus sequence of m^6^A modification has been identified as “RR m^6^AACH” in the nuclear transcriptome of mammals and plants, where R is A/G and H is A/C/U [11].

In *A. thaliana* chloroplasts, 65% of RNA fragments contained the consensus sequence of “RRm^6^ACH”, and the two most observed motifs were GGm^6^ACC (10.3%) and GGm^6^ACU (10.7%). These motifs’ high degree of homology to those in mammalian and other plant nuclear transcriptomes suggests evolutionary conservation [11]. Among the extensively methylated transcripts of chloroplast genes, ribosomal RNA and tRNA were present, as well as numerous transcripts encoding proteins such as translocon at the inner envelope membrane of chloroplasts YCF1.2 (ATCG01130); YCF5—cytochrome C assembly protein (ATCG01040); ATPH—chloroplastic ATP synthase subunit c (ATCG00140); NDHF—NADH dehydrogenase unit (ATCG01010); PSBJ—photosystem II core protein J (ATCG00550); and PSAI—subunit of photosystem I core (ATCG00510) [11].

Current evidence highlights the crucial role of m^6^A modification in plant growth and stress response, with chloroplasts serving as the principal sensors of environmental fluctuations. However, despite significant progress, our understanding of chloroplast methyltransferases, demethylases, and RNA-binding proteins remains incomplete. Notably, the PFC1 methyltransferase was identified as the primary m^6^A writer responsible for adenine methylation of rRNA in chloroplasts. Experimental observations indicate that four additional S-adenosyl-L-methionine-dependent methyltransferases are localized in chloroplasts. These methyltransferases are encoded by the genes At1g78140, At2g41040, At4g29590, and At5g63100 [12,13]. Furthermore, predictive models, based on sequence similarity, indicate the probable chloroplast localization of two more S-adenosyl-L-methionine-dependent methyltransferases, encoded by At5g44490 and At5g44600 [13,14]. However, the function of these enzymes remains unknown.

In addition to N6-methyladenosine (m^6^A) RNA methylation, chloroplasts are also subject to other types of RNA methylation modifications, namely, N4-cytosine methylation leading to the formation of N4-methylcytidine (m^4^C) and N2-guanine methylation resulting in the formation of 2-methylguanosine (m^2^G). These modifications are associated explicitly with 16S rRNA [10,15]. Little is known about the enzymes involved in these processes. In *A*. *thaliana*, Chloroplast MraW-Like (CMAL) was found to be responsible for the m^4^C methylation of C1352 in chloroplast 16S rRNA, impacting ribosome biogenesis, plant development, and hormonal responses [15].

### 2.2. Enzymes Involved in Chloroplast RNA Methylation

#### 2.2.1. PFC1

The *PFC1* gene encodes a methyltransferase crucial for regulating chlorophyll production and is involved in plant response to temperature stress. Under chilling and rewarming conditions, mutants lacking PFC1 display distinct phenotypic changes. The *A. thaliana* plants were grown at a standard temperature of 22 °C for two weeks and then transferred to 5 °C for 5 months. The leaves that developed at 22 °C remained green and photosynthetically active in low-temperature conditions; however, their photosynthetic activity was reduced. The leaves that initially developed at 22° C and 5 °C displayed chimeric pigmentation; i.e., the tips of the leaves remained green, while chlorosis appeared in the basal parts of the leaves. The leaves that developed entirely at 5 °C were fully chlorotic but contained carotenoids. After 5 months of exposure to low-temperature stress, plants were transferred to 22 °C, and green tissue began to develop at the leaf bases of immature chlorotic leaves, which is typical for dicot leaves, the maturation of which progresses from tip to base. The green leaves that developed during the initial 22 °C condition and the entirely chlorotic leaves had senesced, and all of the new tissues grown at 22 °C were green [16]. The molecular mechanism underlying these changes is poorly understood. It has been shown that in a *pfc1* mutant plant, two adenosines at the 3′ end of chloroplast 16S rRNA remain unmethylated [16]. One hypothesis suggests that the absence of PFC1 protein destabilizes a 16S rRNA–protein complex in a temperature-dependent manner. Alternatively, the lack of methylation might interfere with pre-rRNA processing or a subsequent step in ribosome assembly.

#### 2.2.2. CMAL

Chloroplast MraW-Like (CMAL) is a methyltransferase incorporating m^4^C in 16S rRNA. Experimental evidence confirms CMAL’s localization within chloroplasts, where it associates specifically with chloroplast nucleoids [15]. Notably, deficiencies in CMAL lead to disruptions in the accumulation of ribosomal subunits, thereby affecting the biogenesis and function of ribosomes. In turn, this leads to reduced chloroplast translation in *A. thaliana cmal* mutants that negatively impact the biosynthesis of Trp and the production of indole-3-acetic acid (IAA) [15]. Given CMAL’s essential role in chloroplast functioning, the overall development of plants is significantly impacted by its activity. *A. thaliana cmal* mutants exhibit a high chlorophyll fluorescence phenotype and a reduced F*_v_*/F*_m_* ratio in emerging leaves, which are attributed to impaired thylakoid development. Additionally, these mutants display various developmental abnormalities, including narrow leaf blades, retarded root growth, impaired cell expansion, and reduced starch levels, all of which suggest impaired amyloplast development [15].

#### 2.2.3. RSMD

RSMD is an ortholog of *E. coli* 16S methyltransferase. In *A. thaliana,* the protein is nuclear-encoded (At3g28460). However, its chloroplast localization has been experimentally confirmed. RSMD was also proven to be responsible for m^2^G methylation at position 915 in 16S rRNA [17]. Under standard laboratory conditions, mutants lacking RSMD exhibited notably reduced photosynthetic efficiency due to reduced levels of photosystem II proteins, including proteins D1 and D2, which form the photosystem reaction center, as well as CP43 and CP47, which are essential components of the inner PSII antenna.

Additionally, decreased ATPase subunit ATPF content was observed. Moreover, RSMD appeared to play a significant role in the response to cold stress. Cold treatment of *rsmd* mutants resulted in slower growth, shorter roots, and pale green pigmentation of leaves. Notably, these phenotypes are not observed in response to other stresses, such as drought or salt stress [17]. The *A. thaliana rsmd* mutant also showed increased sensitivity to lincomycin, an inhibitor of chloroplast translation. This effect was intensified under cold stress conditions, affecting the creation of chloroplast ribosomes [10]. It can thus be concluded that RSMD methyltransferase plays a crucial role in proper chloroplast development and functioning.

#### 2.2.4. CP31A

CP31A belongs to a family of nuclear-encoded proteins called chloroplast ribonucleoproteins (cpRNPs). cpRNPs share a common feature: an acidic domain (AD) positioned N-terminally to two RNA-recognition motifs (RRM), critical for interacting with target RNA molecules. CP31A, a key player in chloroplast RNA metabolism, exerts a multifaceted role crucial for maintaining chloroplast function under varying conditions. Firstly, it facilitates RNA editing at 13 specific sites within chloroplast transcripts, ensuring precise gene expression and functional protein production [18,19]. Additionally, CP31A contributes to the accumulation of various chloroplast mRNAs, especially in increasing the quantity of mRNA encoding an NDH complex subunit F [20]. The role of CP31A becomes crucial for proper chloroplast development and functionality under low-temperature stress [21]. *A. thaliana* mutants lacking the CP31A protein show sensitivity to cold temperatures, resulting in lower germination rates and in *tissue bleaching* in emerging leaves at 8 °C. CP31A *also contributes* to chloroplast development by stabilizing RNA. It binds to the common 3′-terminus of RNAs, protecting them from degradation by 3′-exonucleases [21]*,* and the acidic domain of CP31A was proven essential for proper chloroplast development in cold conditions [19].

#### 2.2.5. CP33A

CP33A, another member of the cpRNPs family, plays a pivotal role in chloroplast RNA metabolism and plant development. It functions globally in processing and stabilizing chloroplast RNAs. CP33A associates with all chloroplast mRNAs, particularly unspliced and unprocessed precursor mRNAs, contributing significantly to their stabilization. Interestingly, CP33A defines a ribodomain separate from the chloroplast translation machinery, suggesting its distinct role in RNA processes within the chloroplast [22]. CP33A absence leads to albino seedlings with abnormal leaf development, indicating its crucial role in chloroplast biogenesis. These seedlings can only survive with an external carbon source, highlighting the severity of CP33A loss. Overall, CP33A’s multifaceted functions are indispensable for chloroplast RNA stability and essential processes in higher plant chloroplasts, thus influencing overall plant development [22].

### 2.3. Chloroplast RNA Pseudouridylation

Pseudouridylation, the process of converting uridine (U) into pseudouridine (Ψ), is another post-transcriptional RNA modification occurring in chloroplasts, which was proven to participate in the regulation of gene expression. Due to its common occurrence and evolutionary conservation in all living organisms, pseudouridine is considered the fifth ribonucleoside [23]. Experimental data accumulated thus far have indicated that Ψ is present in many different types of RNAs, including coding and noncoding RNAs. Ψ is particularly abundant in rRNA, spliceosomal snRNAs, and mRNA [23,24]. Pseudouridine was shown to influence mRNA stabilization by reducing the binding of proteins to their target mRNAs [25]. In the *A. thaliana* genome, hundreds of pseudouridine sites were identified in noncoding RNAs and mRNA [8]. It was also shown that Ψs preferentially occur in the coding sequences and 5′-UTR of mRNAs in *A. thaliana* and that UUC, CUU, UUU, and UCU triplet codons were the most frequently pseudouridylated. Interestingly, the first U in a codon tended to have a higher Ψ occurrence than the second U, except for in the case of UUU [8]. Two different mechanisms of pseudouridine synthesis have been identified. The first involves pseudouridine synthases (PUSs) catalyzing the isomerization of U to Ψ on their own. The second mechanism involves ribonucleoproteins (RNPs) pseudouridylating U. However, the process of pseudouridylation in prokaryotic RNAs relies exclusively on the activity of PUSs. Pseudouridylation occurs also in chloroplast RNA. The first evidence of pseudouridylation in chloroplasts was demonstrated in vitro for tRNA [26]. Interestingly, the reaction’s activity was strongly temperature-dependent and occurred at 25 °C but not at 37 °C. According to the TAIR database (accessed March 2024) [14], 20 genes encoding potential PUSs were identified in the *A. thaliana* genome, and 10 of their products were assigned as localized in the chloroplast. So far, chloroplast localization of two of them has been confirmed experimentally, namely, RLUA4 (At1g56345) and SVR1 (At2g39140) [27]. The function of RLUA4 remains uninvestigated. Two additional pseudouridine synthases, TCD3 and OsPUS1, were identified in rice chloroplasts [9,28].

### 2.4. Enzymes Involved in Chloroplast RNA Pseudouridylation

#### 2.4.1. SVR1

SVR1 was found to play an important role in maintaining proper RNA-related processes in chloroplasts. It has been demonstrated that *svr1 A. thaliana* mutants exhibit defects in chloroplast rRNA processing and translation, characterized by a reduced abundance of chloroplast proteins and elevated levels of chloroplast mRNA [29]. Among the chloroplast proteins with reduced accumulation levels, 53 were ribosomal proteins, and 63 were related to photosynthesis. Among the ribosomal proteins with altered accumulation, several were from the large ribosomal subunit, including RPL35, RPL36, RPL28, RPL27, RPL2-A, and RPL9. Among the less abundant photosynthesis proteins, eight from photosystem I (PSAA-H) and six from photosystem II (PSBA-F and PSBL) were identified, along with the ribulose bisphosphate carboxylase large chain (RBCL), ribulose bisphosphate carboxylase small chains 1A and 1B, and the iron–sulfur subunit PETC [8]. At the phenotypic level, the *svr1* mutants exhibited significantly smaller rosette sizes. Furthermore, s*vr1* mutants also showed decreased sensitivity to a deficiency of inorganic phosphate (Pi) [30].

#### 2.4.2. TCD3

TCD3 is chloroplast-localized Ψ synthase. In rice, TCD3 was crucial for chloroplast development at a lower temperature [9]. When cultivated at 20 °C, the *tcd3* mutant exhibited yellowish-white leaves. Photosynthetic pigments chlorophyll a and b, as well as carotenoids, were undetectable, and no properly developed chloroplasts were observed. The plants died after the five-leaf stage. At a temperature of 20 °C, the disruption of TCD3 also resulted in a significant decrease in the transcript levels of chloroplast-associated genes and ribosomal genes involved in chloroplast rRNA assembly. However, at 32 °C, the transcript levels of these genes did not change, and the *tcd3* rice mutants grew normally [9], suggesting that TCD3 may play a direct or indirect role in the processing of chloroplast rRNA.

#### 2.4.3. OsPUS1

OsPUS1 is essential for the pseudouridylation of chloroplast rRNA, a modification necessary for the biogenesis and function of chloroplast ribosomes [28]. The enzyme’s pseudouridylation activity ensures that rRNA maintains its proper structure, facilitating the correct assembly of ribosomal subunits. This process is particularly critical under low-temperature conditions, where the demand for functional ribosomes increases. In the *ospus1-1* mutant, which lacks functional OsPUS1, the absence of this enzyme leads to temperature-dependent phenotypes [28]. At low temperatures, these mutants exhibit an albino phenotype characterized by a lack of chlorophyll and impaired chloroplast development due to the failure in ribosome biogenesis and function, which hampers the translation of essential chloroplast-encoded proteins. However, at standard temperatures, the phenotype is less severe, suggesting that higher temperatures can partially compensate for the loss of OsPUS1 by facilitating rRNA maturation and ribosome assembly. Additionally, OsPUS1 is involved in a complex communication network between the nucleus and chloroplasts, coordinating the plant’s response to environmental stress [28]. Under stress conditions, defective ribosomes in *ospus1-1* mutants lead to increased ROS levels, which act as retrograde signals to the nucleus. This signaling prompts the nucleus to adjust gene expression, enhancing stress resilience and maintaining cellular homeostasis. Through this mechanism, OsPUS1 helps plants adapt to and survive in adverse environmental conditions.

## 3. Extrachloroplastic RNA Modifications Influencing Chloroplast Functioning and Photosynthesis Performance

### 3.1. Extrachloroplastic RNA Methylation

Chloroplasts depend on proteins encoded by both their own genome and the nuclear genome, requiring precise coordination between these genetic systems. Cytoplasmic RNA modifications are essential in modulating this coordination, ensuring that chloroplasts can efficiently perform photosynthesis, produce energy, and synthesize essential compounds. The dynamic nature of RNA modifications allows plants to respond swiftly to environmental changes, enhancing their resilience and adaptability. In the A. thaliana, the functional m^6^A methyltransferase complex is formed by mRNA adenosine methylase (MTA), MTB, FKBP12-interacting protein 37KD (FIP37), VIRILIZER (VIR), HAKAI, and HAKAI-interacting zinc finger protein 2 (HIZ2) [31]. All of these components, except HAKAI, are essential for embryo development. Depletion of MTA, MTB, FIP37, or VIR results in embryo arrestment at the globular stage of seed development [32]. During postembryonic development, MTA- and FIP37-mediated m^6^A methylation occurs on the mRNAs of two key meristem regulators. VIR, MTB, and HIZ2 downregulation affects shoot development and root growth [31,32]. In addition to developmental processes, m^6^A methylation is also linked to biotic and abiotic stress responses in plants and to the maintenance of photosynthetic efficiency in changing environmental conditions.

### 3.2. Enzymes Involved in Extrachloroplastic RNA Methylation

#### 3.2.1. FIP37

FIP37 is part of the functional m^6^A methyltransferase complex formed by mRNA adenosine methylase and was proven to play a crucial role in acclimating to cold stress and maintaining efficient photosynthesis at low temperatures. While, under standard conditions, *A. thaliana fip37* mutants do not show significant impairment in the maximum quantum yield of PSII, their response to cold treatment reveals a vital function of this gene. During cold exposure, the Fv/Fm ratio—a key indicator of photosynthetic performance—declines significantly faster in *A. thalina fip37* mutants compared to in wild-type plants, suggesting that FIP37 is involved in stabilizing the photosynthetic machinery during cold acclimation. Despite the rapid decline during cold stress, the full recovery of the Fv/Fm ratio in *fip37* mutants upon return to normal temperatures indicates that FIP37’s role is crucial in providing resilience to cold-induced stress rather than in regulating photosynthesis under standard conditions [33]. A closer examination revealed that under cold stress conditions, the *fip37* mutant exhibited a reduction in chlorophyll content along with alterations in chlorophyll *a* fluorescence parameters. Basal non-photochemical energy loss in PSII increased during cold treatment, indicating elevated oxidative stress. Simultaneously, significant photosystem I activity was decreased, primarily due to limitations on the donor side, suggesting reduced electron flow toward PSI [33]. Based on these observations, it was suggested that during cold treatment, the photosynthetic electron transport in *fip37* mutants is significantly affected, leading to increased oxidative damage and reduced PSI. After a 4-day cold treatment, the mutant exhibited a more pronounced decrease in the protein levels of PSI (PSAA, PSAO), the Cyt_b6f_ complex, and CET (PGRL1, NDHB). Interestingly, the levels of PSII, ATP synthase proteins, and PGR5 remained relatively stable in both the cold-treated WT and mutant plants. Notably, CURT1, crucial for thylakoid membrane curvature [34], also decreased after exposure to the 4 °C condition, which led to ultrastructural changes in chloroplasts, such as the wavy organization of thylakoids and fewer membrane layers in grana stacks [33]. The observed differences in protein abundance did not correlate with changes in gene expression, indicating that they resulted from events occurring at the post-transcriptional level. It was also found that the cytoplasmic translation of RNAs responsible for chloroplast proteins likely contributes to the observed deficiencies in plastid proteins in *fip37*. Furthermore, the stability of protein biosynthesis within chloroplasts implies that the decrease in plastid-encoded proteins may result from their instability, which is attributed to the diminished presence of specific nuclear-encoded chloroplast proteins that are essential for forming photosynthetic complexes [33].

#### 3.2.2. VIR

It was found that VIRILIZER1 (VIR), a protein that is part of the m^6^A writer complex, is crucial for maintaining photosynthetic efficiency under high-light stress. The *vir* mutants were found to be highly sensitive to high light (HL) [35]. Under standard lighting conditions, there was no difference in the maximum photochemical efficiency of PSII (Fv/Fm) between *vir* mutants and wild-type plants. However, *vir* mutants displayed lower photochemical quenching (qP), indicating a reduced ability to convert light efficiently in their photochemical processes. After 4h of exposure to high light, the Fv/Fm values in *vir* mutants were significantly lower than those in wild-type plants. Additionally, non-photochemical quenching (NPQ) values increased at a slower rate in *vir* mutants than in wild-type plants, suggesting a delayed response in dissipating excess energy. The Fv/Fm values in *vir* mutants recovered to levels similar to those in wild-type plants after 2 days under standard light conditions. The introduction of a functional VIR gene rescued the high-light-sensitive phenotype. Further analysis revealed significantly reduced accumulation levels of proteins related to photoprotection (HHL1, MPH1, HCF244, DEG1, and STN8) in the *vir* mutant compared to wild-type plants, both before and after high-light (HL) treatments. It was also shown that VIR influences mRNA stability. In *vir* mutants, the mRNAs for essential PSII repair genes (HHL1 and MPH1) as well as for other photoprotection-related genes were degraded more rapidly than in the wild-type, suggesting that lower m^6^A levels promote mRNA degradation. It has been established that VIR directly binds to the mRNAs of photoprotection-related genes such as HHL1, MPH1, HCF244, DEG1, cpTATC, and STN8, suggesting that it plays a role in stabilizing or translating these mRNAs. Additionally, 182 VIR-associated proteins were identified, including HAKAI (an m^6^A writer); key players in RNA stability and translation, such as EIF3E, RNJ, RH3, and RH6; proteins related to chloroplast gene expression, including PTAC4, PTAC5, PTAC16; and RRM-containing proteins UBA2C and ATG3BP6. This indicates that the increased sensitivity to high light in the *vir* mutant may result from the combined effects of multiple target genes and that VIR may serve as a central hub in mediating a complex photoprotection network through various post-transcriptional processes, similar to the regulatory role of LONG HYPOCOTYL5 (HY5) in transcriptional regulation [35].

#### 3.2.3. ALKBH10B

Alpha-ketoglutarate-dependent dioxygenase homolog 10B (ALKBH10B), an m^6^A RNA demethylase, has a pivotal role in controlling the intricate dynamics of plant growth [36] and the flowering transition [37]. The loss of function in ALKBH10B resulted in delayed flowering, indicating that flowering time depends on the catalytic activity of ALKBH10 [37]. Additionally, ALKBH10B was found to participate in abiotic stress responses. In *A. thaliana*, the expression of ALKBH10B is increased under osmotic and salt stress [38]. The *alkbh10b* mutants showed hypersensitivity to osmotic and salt stress during seed germination [36]. Other research indicates, however, that although germination of *alkbh10b* mutant seeds was markedly delayed under salt stress, the seedling growth and survival rate were enhanced [39]. In *alkbh10b* mutants, a reduction in the expression levels of genes encoding negative effectors of salt stress, including ATAF1, BGLU22, and MYB7, was also observed [39]. The induced expression in response to salt stress was also detected in *Populus* sp. [40]. Conversely, GhALKBH10B is downregulated in cotton roots under salt stress conditions [41]. Also, in salt-tolerant cotton varieties, a lower expression of the gene was observed. In tomato *alkbh10b* mutants, the response to salt stress leads to significant improvements in water retention, increased accumulation of photosynthetic products and proline, and diminished levels of reactive oxygen species, thereby lowering cellular damage. Similar effects were observed in response to drought stress, indicating the role of the demethylase in regulating the response to a water deficit [42]. These observations are consistent with the effects of silencing ALKBH10B in cotton plants, which led to enhanced seedling growth and survival rates under drought stress. This increase correlates with higher mRNA stability of genes related to photosynthesis such as the gene GhSnRK2;3, which encodes an important regulator in ABA-dependent signaling [43]. However, in *A. thaliana* mutants, a lack of ALKBH10B demethylase led to a higher sensitivity of plants to drought stress, while its overexpression increased their resistance to this stress [44]. Hence, the function of ALKBH10B demethylase in governing salt and drought tolerance varies across plant species and development stages. The molecular mechanism of ALKBH10B’s action is related to ABA signaling [36,43]. The mutation in this gene influences plant sensitivity to ABA and modulates gene expression within the ABA-dependent signaling pathway. ABA induces ALKBH10B expression, but this inducibility is lost in the *abi1-1* mutant. The *abi1-1* mutation results in plants insensitive to ABA, primarily due to a loss of function in the phosphatase enzyme ABI1, which regulates ABA signaling [45]. These findings suggest that ALKBH10B is regulated by ABA signaling and acts downstream of ABI1 phosphatase. Furthermore, RNA-seq data indicate that ABA response genes, including the transcription factors ABI3 and ABI5—known positive regulators of ABA signaling during seed germination—are upregulated in *alkbh10b* following ABA treatment [36]. It was also found that few ABA response genes are m^6^A-hypermethylated in *alkbh10b,* indicating a role for ALKBH10B-mediated m^6^A demethylation in ABA signaling gene regulation. Among these hypermethylated genes, PYR1, PYL4, PYL7, PYL9, and ABI1 were found [36].

#### 3.2.4. OsNSUN2

OsNSUN2 is an RNA 5-methylcytosine (m^5^C) methyltransferase identified in rice as an enzyme localized in the nucleus and crucial for maintaining chloroplast functioning under heat stress conditions. OsNSUN2-mediated m^5^C modification is significant as the enzyme impacts approximately one-third of mRNAs encoding chloroplast. In response to heat stress, the methylation of 60 sites was induced in an OsNSUN2-dependent manner (of which 53 were mRNA sites). The m^5^C modification was found to be especially strongly induced in four mRNAs, those encoding OsPAL1 (phenylalanine ammonia-lyase 1), β-OsLCY (lycopene β-cyclase), OsGLYI4 (glyoxalase I 4), and OsPRX55 (peroxidase 55). This induction was accompanied by increased protein synthesis. OsPAL1 is involved in the phenylpropanoid pathway, which produces a variety of stress-related secondary metabolites, such as anthocyanins and lignin. β-OsLCY participates in carotenoid biosynthesis necessary for maintaining proper photosynthesis efficiency. OsGLYI4 is involved in the detoxification of the cytotoxic molecule methylglyoxal (MG), and OsPRX55 is crucial for managing reactive oxygen species in cells [46]. It was found that OsNSUN2 ensures the prioritized translation of genes essential for adaptation and survival under heat stress conditions. Moreover, this modification enhances translation efficiency, allowing for the rapid synthesis of proteins necessary for maintaining cellular homeostasis. The dysregulation of OsNSUN2-dependent mRNAs compromises the plant’s ability to adapt to heat treatment effectively [46]. OsNSUN2 is also involved in the expression of genes related to pigment synthesis and photosynthetic apparatus assembly [46]. Under heat stress, *osnsun2* mutants exhibit a significant decrease in chlorophyll and carotenoid contents. Further analysis of *osnun2* mutants revealed a downregulation of enzymes necessary for chlorophyll biosynthesis and degradation, potentially leading to the accumulation of phototoxic chlorophyll metabolites. Dysfunctional chlorophyll degradation and reduced levels of carotenoid pigments were also observed, suggesting a compromised photoprotective mechanism. Chlorophyll fluorescence imaging revealed that during heat treatment of *osnsun2*, the maximal PS II quantum yield was lower, and the PS II quantum yield was effective. Simultaneously, excessive reactive oxygen species (ROS) production was observed. Overall, the absence of OsNSUN2 disrupts the adaptation process, leading to a decline in photosynthesis after prolonged exposure to heat. Obtained results indicate a connection between OsNSUN2-dependent mRNA m^5^C modification, photosystem function, and chlorophyll metabolism.

## 4. Conclusions

**Figure 1:**
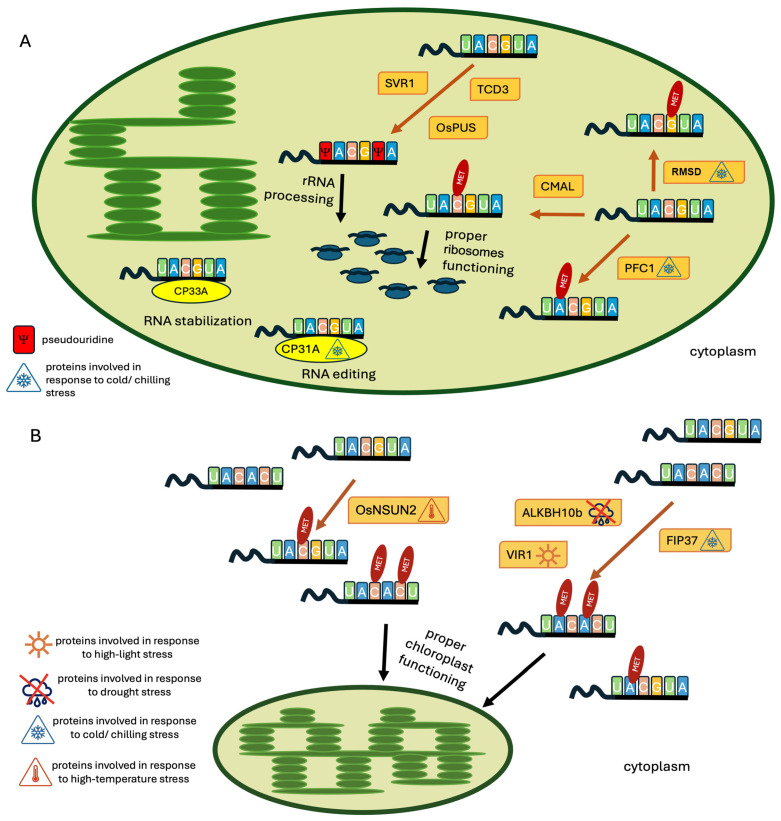
Chloroplast-(**A**) and extrachloroplast (**B**) -located proteins involved in RNA modification and participating in the maintenance of proper chloroplast function.

RNA modifications in plants are vital for controlling numerous cellular processes, offering a nuanced layer of gene regulation. These modifications, such as N6-methyladenosine (m^6^A) and 5-methylcytosine (m^5^C), play critical roles in plant development, including in organ and root formation and flower development [47]. Alterations in methylation machinery can lead to significant developmental defects, such as disrupted vascular tissue development, root growth, and flower architecture. The effects of methylation depend on the reader proteins, often members of the YTH family, which bind to methylated mRNA and influence its metabolism by enhancing mRNA stability, translation, and nuclear export. Methylation is essential for regulating gene expression in plants, impacting development and stress responses by modulating the stability and translation of specific transcripts [47]. Pseudouridylation enhances the stability and function of tRNAs and rRNAs, which are crucial for efficient protein synthesis and help maintain the proper functioning of ribosomes and other components of protein synthesis machinery, ensuring that biosynthetic enzymes are correctly produced [48]. Modifications of chloroplast and nuclear/cytoplasmic RNA significantly impact chloroplast function, photosynthesis efficiency, and the regulation of responses to environmental stimuli. Two crucial RNA modifications, N6-methyladenosine (m^6^A) and 5-methylcytosine (m^5^C), play vital roles in RNA stability, processing, and translation, extending their regulatory influence into chloroplasts. Enzymes such as PFC1, CMAL, and RSMD are necessary for chloroplast RNA methylation (Figure 1A), affecting chloroplast function and development. Pseudouridylation is another critical modification for chloroplast rRNA processing and translation, with enzymes like SVR1, TCD3, and OsPUS1 playing vital roles (Figure 1A). RNA modifications outside of chloroplasts that are known to influence chloroplast function are methylations forming m^6^A and m^5^C, with the involvement of enzymes such as FIP37, VIR1, ALKBH10B, and OsNSUN2 (Figure 1B), which help stabilize photosynthesis and respond to stress conditions. The list of proteins involved in regulating chloroplast function via RNA modification is shown in Table 1. The molecular mechanisms and physiological importance of these events are not yet well understood. It is critical to conduct further research on the influence of RNA modifications on chloroplast function. Understanding how modifications like N6-methyladenosine (m^6^A) and 5-methylcytosine (m^5^C) regulate chloroplast gene expression can enhance photosynthetic efficiency, essential for improving crop yields and ensuring food security. These modifications also significantly affect plant responses to environmental stresses, making crops more resilient to climate change.

## Figures and Tables

**Table 1:** Proteins involved in RNA modification and participating in the maintenance of proper chloroplast function.

Chloroplast-Located Proteins Involved in RNA Modification and Participating in the Maintenance of Proper Chloroplast Function			
METHYLATION			
Name	RNA Modification	Activity	Phenotype
PFC1	m^6^A	adenine demethylase	chlorotic phenotype during chilling stress
CMAL	m^4^C	methylase	affects biogenesis and function of ribosomes
CP31	---	RNA-binding protein	participates in cold stress response, RNA editing
CP33	---	RNA-binding protein	chloroplast development, RNA stabilization
RSMD	m^2^G	methyltransferase	participates in cold stress response
**PSEUDOURIDYLATION**			
TCD3	Ψ	pseudouridine synthase	temperature-sensitive chloroplast development and pigment biosynthesis
SVR1	Ψ	pseudouridine synthase	chloroplast rRNA processing and translation
OsPUS	Ψ	pseudouridine synthase	chloroplast rRNA processing and translation; temperature-sensitive chloroplast development
**Extrachloroplast-Located Proteins Involved in RNA Methylation and Participating in the Maintenance of Proper Chloroplast Function**			
FIP37	m^6^A	subunit of adenine demethylase	stabilizes photosynthesis during cold stress
VIR1	m^6^A	subunit of adenine demethylase	participates in high-light stress response
ALKBH10b	m^6^A	demethylase	involved in salt and drought stress response
OsNSUN2	m^5^C	methyltransferase	participates in heat stress response

## Data Availability

No new data were created or analyzed in this study.
